# Supplementary material for: And the credit goes to … - Ghost and honorary authorship among social scientists
Source: PLoS One. 2022 May 5;17(5):e0267312. doi: 10.1371/journal.pone.0267312 (PMC9070929; doi:10.1371/journal.pone.0267312)
Supplement: S1 Table — (PDF) [file pone.0267312.s001.pdf]

Supporting Information for “And the Credit Goes to … - Ghost and Honorary Authorship among Social Scientists”

S2 Table. Descriptive statistics for dichotomous variables.

	Number	Share
All Respondents	2222	100,00%
Female	738	33,21%
Anglophone (British Isles, North America, Australia & NZ)	866	38,97%
Continental Europe	900	40,50%
Developing Countries (Latin America, Africa, Southeast	300	13,50%
PhD Students	204	9,18%
Professors	1156	52,03%
Editors	541	24,35%
Business Researchers	789	35,51%
Economics and Finance Researchers	291	13,10%
Computer, Operations and Statistics Researcher	352	15,84%
Political Scientists	214	9,63%
Psychologists	136	6,12%
Sociologists	184	8,28%
