# Supplementary material for: And the credit goes to … - Ghost and honorary authorship among social scientists
Source: PLoS One. 2022 May 5;17(5):e0267312. doi: 10.1371/journal.pone.0267312 (PMC9070929; doi:10.1371/journal.pone.0267312)
Supplement: S2 Table — (PDF) [file pone.0267312.s002.pdf]

Supporting Information for "And the Credit Goes to … - Ghost and Honorary Authorship among Social Scientists"

S3 Table. Descriptive statistics for integer variables.

	N	Mean	SD	Minimum	Maximum
Age	2215	46.52	13.03	21	93
Academic Working Years	2222	17.46	12.49	0	63
Papers Published	2222	6.40	7.74	0	60
Reviews Written	2222	6.84	9.37	0	60
# Authors in Last Paper	2052	2.83	1.25	1	6
# Contributors in Last Paper	2052	1.16	1.64	0	6
