# Supplementary material for: And the credit goes to … - Ghost and honorary authorship among social scientists
Source: PLoS One. 2022 May 5;17(5):e0267312. doi: 10.1371/journal.pone.0267312 (PMC9070929; doi:10.1371/journal.pone.0267312)
Supplement: S3 Table — (PDF) [file pone.0267312.s003.pdf]

Supporting Information for “And the Credit Goes to … - Ghost and Honorary Authorship among Social Scientists”

S4 Table. Pairwise correlations coefficients for all employed variables.

	1	2	3	4	5	6	7	8	9	10	11	12	13	14
	Empirical Study						Vignette 1			Vignette 2			Vignette 3	
	Ghost	Honor.	# of Ghost Authors	# of Hono.	Perc. Ghost Authors	Perc. Honor. Authors	Prof.	Postd.	SA	Prof./ Postd.	Prof.	SA	Prof.	PhD
Group							-0.0042	0.0073	0.0098	-0.0046	0.0844	0.0094	-0.0011	-0.0371
Female	-0.0167	0.0569	-0.0154	0.0685	0.0262	0.0507	-0.0095	-0.0040	-0.0127	-0.0370	-0.0200	-0.0337	-0.0214	0.0090
Anglophone	-0.0708	-0.1053	-0.0563	-0.0886	-0.0580	-0.0244	0.0805	0.0666	-0.0969	0.0222	0.0780	-0.0694	0.0566	-0.0157
Continental	-0.0526	-0.0550	-0.0484	-0.0529	-0.0472	-0.0537	-0.0170	0.0263	0.0331	0.0205	-0.1251	0.0076	0.0250	-0.0110
Developing	0.1298	0.1615	0.1084	0.1297	0.1068	0.0488	-0.0966	-0.0404	0.1032	-0.0443	0.0161	0.0897	-0.0996	0.0039
Age	0.0179	-0.0677	0.0052	-0.0857	-0.0243	-0.1142	-0.0493	0.0002	0.0359	0.0185	0.0388	0.0153	0.0033	0.0105
PhDStudent	0.0461	0.0811	0.0517	0.0985	0.0245	0.1019	-0.0101	-0.0505	0.0092	-0.0496	0.0032	-0.0160	-0.0186	-0.0068
Professor	-0.0027	-0.0830	-0.0000	-0.1051	-0.0431	-0.1181	0.0175	-0.0095	-0.0078	0.0069	0.0186	0.0096	0.0038	0.0077
Editor	0.0327	0.0038	0.0250	0.0147	0.0058	-0.0174	-0.0684	-0.0549	0.0272	-0.0093	0.0486	0.0534	0.0025	0.0148
Working Years	0.0048	-0.0884	0.0044	-0.1002	-0.0384	-0.1267	0.0018	-0.0033	0.0383	0.0176	0.0454	0.0226	0.0216	0.0009
Published	0.0052	0.0262	-0.0115	0.0249	0.0014	-0.0181	0.0269	-0.0468	0.0577	-0.0167	0.0937	0.0929	-0.0200	0.0895
Reviews	-0.0142	-0.0404	-0.0181	-0.0514	-0.0691	-0.0644	0.0777	-0.0051	-0.0255	0.0403	0.0546	-0.0150	0.0484	0.0658
Business	-0.0340	0.0412	-0.0285	0.0253	0.0417	0.0511	0.0028	0.0503	-0.1497	-0.0356	0.0112	-0.0934	-0.0204	0.0496
Economics	-0.0077	-0.0916	-0.0215	-0.0988	-0.0086	-0.0511	0.0462	0.0243	-0.0575	0.0236	-0.0207	0.0212	0.0236	-0.0317
IT & OS	0.0595	0.0829	0.0683	0.0740	-0.0373	-0.0054	0.0322	-0.0677	0.1780	0.0422	0.1282	0.1559	0.0274	0.1056
Political	-0.0397	-0.0900	-0.0412	-0.0936	-0.0131	-0.0719	-0.0501	-0.0331	-0.0213	-0.0043	-0.1570	-0.0538	0.0016	-0.1666
Psychology	-0.0091	0.0347	0.0022	0.0796	-0.0036	0.0327	0.0173	0.0151	-0.0226	0.0384	0.1031	-0.0635	0.0542	0.1012
Sociology	-0.0086	-0.0481	-0.0020	-0.0384	0.0064	-0.0075	-0.0138	0.0218	0.0298	0.0007	-0.1097	0.0047	-0.0110	-0.1043

	15	16	17	18	19	20	21	22	23	24	25	26	27	28
Group	1.0000													
Female	0.0260	1.0000												
Anglophone	-0.0126	-0.0329	1.0000											
Continental	0.0005	0.0374	-0.6643	1.0000										
Developing	0.0198	0.0096	-0.3148	-0.3293	1.0000									
Age	-0.0035	-0.1445	0.2703	-0.2142	-0.0620	1.0000								
PhDStudent	-0.0060	0.0816	-0.0903	0.0643	0.0387	-0.3264	1.0000							
Professor	0.0162	-0.1259	0.1602	-0.1458	-0.0243	0.5485	-0.3071	1.0000						
Editor	-0.0025	-0.0577	0.0222	-0.0332	0.0035	0.1904	-0.1155	0.2504	1.0000					
Working Years	0.0018	-0.1500	0.2179	-0.1279	-0.1119	0.8665	-0.2958	0.5639	0.2061	1.0000				
Published	-0.0084	-0.0813	-0.0032	0.0043	-0.0163	0.1429	-0.1710	0.2570	0.2876	0.1944	1.0000			
Reviews	-0.0032	-0.1319	0.1607	-0.0527	-0.1508	0.1465	-0.2430	0.3294	0.3059	0.1826	0.4735	1.0000		
Business	0.0028	0.0175	0.2152	-0.1384	-0.1009	0.0376	-0.0055	0.0377	-0.0514	-0.0359	-0.0799	0.0307	1.0000	
Economics	0.0033	-0.0891	-0.0809	0.0698	0.0175	-0.0194	-0.0372	0.0336	0.0151	0.0117	-0.0286	0.0070	-0.2926	1.0000
IT & OS	0.0111	-0.0641	-0.1323	0.0098	0.1249	-0.0208	0.0147	0.0189	0.0053	0.0184	0.0382	-0.0114	-0.3209	-0.1678
Political	-0.0109	-0.0211	-0.0317	0.0560	-0.0110	-0.0716	0.0508	-0.0674	-0.0637	-0.0572	-0.0668	-0.0540	-0.2449	-0.1281
Psychology	0.0038	0.0783	0.0785	-0.0387	-0.0402	0.0109	0.0192	0.0168	0.0437	0.0418	0.1360	0.0566	-0.1934	-0.1011
Sociology	-0.0001	0.0643	-0.0807	0.1286	-0.0492	-0.0144	-0.0253	-0.0442	0.0098	0.0214	-0.0062	-0.0389	-0.2210	-0.1156

	29	30	31	32
IT & OS	1.0000			
Political	-0.1405	1.0000		
Psychology	-0.1109	-0.0846	1.0000	
Sociology	-0.1268	-0.0967	-0.0764	1.0000
