# Supplementary material for: And the credit goes to … - Ghost and honorary authorship among social scientists
Source: PLoS One. 2022 May 5;17(5):e0267312. doi: 10.1371/journal.pone.0267312 (PMC9070929; doi:10.1371/journal.pone.0267312)
Supplement: S4 Table — (PDF) [file pone.0267312.s004.pdf]

Supporting Information for "And the Credit Goes to … - Ghost and Honorary Authorship among Social Scientists"

S5 Table. Regression results of actual and perceived prevalence of ghost and honorary authorship including confidence intervals.

	1<br>Ghost Authorship	2<br>Honorary Authorship	3<br># of Ghost Authors	4<br># of Honorary Authors	5<br>Perceived Ghost Authors	6<br>Perceived Honorary Authors
Rate of Ghost Authors					0.714<br>(0.069)<br>[-0.055,1.483]	
Rate of Honorary Authors						0.935<br>(0.000)<br>[0.590,1.281]
Female	-0.324<br>(0.284)<br>[-0.916,0.268]	0.175<br>(0.099)<br>[-0.033,0.382]	-0.345<br>(0.297)<br>[-0.995,0.304]	0.109<br>(0.118)<br>[-0.028,0.246]	0.151<br>(0.470)<br>[-0.259,0.560]	0.054<br>(0.656)<br>[-0.183,0.290]
Anglophone	-1.619<br>(0.000)<br>[-2.513,-0.724]	-0.964<br>(0.000)<br>[-1.368,-0.560]	-1.416<br>(0.005)<br>[-2.414,-0.418]	-0.612<br>(0.000)<br>[-0.854,-0.370]	-0.663<br>(0.060)<br>[-1.353,0.028]	-0.237<br>(0.250)<br>[-0.641,0.167]
Continental Europe	-1.227<br>(0.003)<br>[-2.048,-0.406]	-0.786<br>(0.000)<br>[-1.183,-0.389]	-1.228<br>(0.013)<br>[-2.194,-0.262]	-0.522<br>(0.000)<br>[-0.759,-0.286]	-0.577<br>(0.087)<br>[-1.238,0.085]	-0.373<br>(0.066)<br>[-0.771,0.025]
Developing Countries	0.259<br>(0.523)<br>[-0.534,1.051]	0.131<br>(0.570)<br>[-0.321,0.584]	0.265<br>(0.608)<br>[-0.747,1.276]	-0.118<br>(0.379)<br>[-0.382,0.145]	-0.085<br>(0.803)<br>[-0.752,0.582]	-0.267<br>(0.240)<br>[-0.712,0.178]
Age	0.031<br>(0.110)<br>[-0.007,0.070]	0.009<br>(0.251)<br>[-0.007,0.025]	0.015<br>(0.534)<br>[-0.032,0.062]	0.006<br>(0.247)<br>[-0.004,0.016]	-0.006<br>(0.738)<br>[-0.039,0.028]	-0.005<br>(0.616)<br>[-0.022,0.013]
Ph.D. Student	1.268<br>(0.004)<br>[0.405,2.130]	0.513<br>(0.012)<br>[0.112,0.914]	1.520<br>(0.004)<br>[0.490,2.550]	0.290<br>(0.0018)<br>[0.050,0.530]	0.012<br>(0.973)<br>[-0.654,0.677]	0.204<br>(0.292)<br>[-0.175,0.583]
Professor	-0.201<br>(0.558)<br>[-0.874,0.472]	-0.287<br>(0.025)<br>[-0.537,-0.036]	0.017<br>(0.965)<br>[-0.740,0.773]	-0.221<br>(0.009)<br>[-0.388,-0.055]	-0.087<br>(0.734)<br>[-0.589,0.415]	-0.171<br>(0.256)<br>[-0.465,0.123]
Editor	0.245<br>(0.424)<br>[-0.356,0.846]	0.067<br>(0.580)<br>[-0.170,0.304]	0.084<br>(0.808)<br>[-0.591,0.759]	0.113<br>(0.158)<br>[-0.044,0.271]	-0.001<br>(0.997)<br>[-0.477,0.475]	0.058<br>(0.686)<br>[-0.222,0.338]
Years in Academia	-0.009<br>(0.659)<br>[-0.050,0.032]	-0.011<br>(0.179)<br>[-0.028,0.005]	0.007<br>(0.779)<br>[-0.040,0.054]	-0.010<br>(0.073)<br>[-0.021,0.001]	0.007<br>(0.685)<br>[-0.027,0.041]	-0.008<br>(0.437)<br>[-0.027,0.012]
Published Papers	-0.023<br>(0.833)	0.074<br>(0.076)	-0.105<br>(0.400)	0.062<br>(0.022)	0.075<br>(0.361)	0.024<br>(0.612)

	[-0.233,0.188]	[-0.008,0.156]	[-0.351,0.140]	[0.009,0.114]	[-0.086,0.236]	[-0.070,0.119]
Written	0.063	-0.010	0.039	-0.020	-0.164	-0.023
Reviews	(0.509)	(0.778)	(0.724)	(0.396)	(0.050)	(0.588)
	[-0.124,0.251]	[-0.080,0.060]	[-0.177,0.255]	[-0.067,0.026]	[-0.328,-0.000]	[-0.105,0.060]
Business	-0.397	0.216	-0.363	0.074	0.181	-0.050
	(0.320)	(0.193)	(0.436)	(0.494)	(0.561)	(0.783)
	[-1.179,0.385]	[-0.109,0.542]	[-1.276,0.551]	[-0.139,0.287]	[-0.429,0.791]	[-0.406,0.306]
Economics	-0.515	-0.430	-0.560	-0.395	-0.110	-0.278
and Finance	(0.304)	(0.031)	(0.349)	(0.004)	(0.783)	(0.237)
	[-1.495,0.466]	[-0.820,-0.040]	[-1.730,0.611]	[-0.667,-0.124]	[-0.892,0.672]	[-0.739,0.183]
Computer	0.056	0.319	0.328	0.150	-0.267	-0.183
and Statistics	(0.891)	(0.090)	(0.499)	(0.212)	(0.504)	(0.389)
	[-0.742,0.854]	[-0.050,0.687]	[-0.623,1.279]	[-0.086,0.386]	[-1.051,0.516]	[-0.601,0.234]
Political Sciences	-0.967	-0.625	-1.294	-0.523	0.129	-0.584
	(0.149)	(0.005)	(0.102)	(0.001)	(0.763)	(0.038)
	[-2.279,0.346]	[-1.059,-0.190]	[-2.846,0.258]	[-0.831,-0.215]	[-0.708,0.966]	[-1.137,-0.032]
Psychology	-0.410	0.321	-0.060	0.338	-0.311	0.056
	(0.545)	(0.182)	(0.930)	(0.024)	(0.571)	(0.828)
	[-1.735,0.916]	[-0.150,0.791]	[-1.419,1.298]	[0.045,0.631]	[-1.384,0.763]	[-0.446,0.557]
Sociology	-0.334	-0.241	0.247	-0.150	0.280	-0.132
	(0.581)	(0.286)	(0.700)	(0.324)	(0.511)	(0.605)
	[-1.519,0.851]	[-0.683,0.201]	[-1.007,1.500]	[-0.448,0.148]	[-0.556,1.117]	[-0.635,0.370]
Chi-Square	55.30	145.69	44.53	140.49	23.68	70.45
P > Chi-Square	0.00	0.00	0.00	0.00	0.1659	0.00
Pseudo R-squared	0.01	0.06	0.06	0.03	0.04	0.04
Observations	1854	1857	1854	1857	804	1818

1 and 2 present marginal effects derived from logistic regressions with p-values in parentheses and 95% confidence intervals in brackets. 3 and 4 present coefficients derived from negative binomial regressions with p-values in parentheses and 95% confidence intervals in brackets. 5 and 6 present coefficients derived from Poisson regressions with p-values in parentheses and 95% confidence intervals in brackets. The number of observations is smaller than 2,222 because we only include respondents who a) published at least one scientific journal article (thus excluding conference participants who already presented but not published papers yet) and b) who did not select N/A in any items relevant for generating the included variables. 1 and 3 analyze ghost authorship whereas 2 and 4 analyze honorary authorship. The difference between 1857 (2 & 4) and 1854 (1 & 3) results from respondents fully answering honorary authorship questions but selecting N/A options in the ghost authorship questions. The difference in 5 between 804 (perceived ghost authors) and 1854 (identified ghost authors) arises from respondents selecting N/A for the sensitive question: “Please indicate on a scale from 0 (disagree) to 100 (agree) that for you last published paper, all researchers who made significant contributions were named as authors.” Moreover, 5 also excludes all respondents stating that there were no non-author contributors to their last published paper. The difference in 6 between 1818 (perceived honorary authors) to 1854 (identified honorary authors) arises from respondents selecting N/A for the sensitive question: “Please indicate on a scale from 0 (disagree) to 100 (agree) that for your last published paper, researchers received authorship only if they participated actively in the creation process.”
