# Supplementary material for: And the credit goes to … - Ghost and honorary authorship among social scientists
Source: PLoS One. 2022 May 5;17(5):e0267312. doi: 10.1371/journal.pone.0267312 (PMC9070929; doi:10.1371/journal.pone.0267312)
Supplement: S5 Table — (PDF) [file pone.0267312.s005.pdf]

Supporting Information for "And the Credit Goes to … - Ghost and Honorary Authorship among Social Scientists"

S6 Table. Regression results of hypothetical authorship assignments in the vignettes including confidence intervals.

	Vignette 1			Vignette 2		
	Professor	Postdoc	Student Assistant	Professor/Postdoc	Professor	Student Assistant
Group	-0.268	0.025	0.012	-0.006	0.429	0.082
	(0.249)	(0.944)	(0.910)	(0.984)	(0.000)	(0.623)
	[-0.724,0.188]	[-0.683,0.734]	[-0.191,0.214]	[-0.597,0.585]	[0.231,0.628]	[-0.245,0.409]
Female	-0.014	-0.411	0.010	-0.133	-0.060	-0.193
	(0.956)	(0.293)	(0.929)	(0.680)	(0.587)	(0.307)
	[-0.498,0.470]	[-1.175,0.354]	[-0.211,0.231]	[-0.761,0.496]	[-0.275,0.156]	[-0.564,0.178]
Anglophone	1.081	2.603	0.044	0.709	-0.195	-0.017
	(0.025)	(0.000)	(0.844)	(0.155)	(0.400)	(0.962)
	[0.137,2.026]	[1.382,3.824]	[-0.397,0.485]	[-0.269,1.687]	[-0.649,0.259]	[-0.706,0.672]
Continental Europe	0.062	1.705	0.299	1.052	-0.772	0.080
	(0.888)	(0.000)	(0.174)	(0.041)	(0.001)	(0.814)
	[-0.794,0.917]	[0.787,2.624]	[-0.132,0.731]	[0.043,2.060]	[-1.217,-0.328]	[-0.588,0.748]
Developing Countries	-0.350	1.051	0.631	0.145	-0.503	0.513
	(0.447)	(0.039)	(0.010)	(0.789)	(0.049)	(0.157)
	[-1.252,0.552]	[0.053,2.049]	[0.154,1.108]	[-0.914,1.203]	[-1.004,-0.003]	[-0.198,1.224]
Age	-0.064	-0.027	0.011	0.008	0.001	0.006
	(0.000)	(0.375)	(0.179)	(0.759)	(0.936)	(0.652)
	[-0.093,-0.034]	[-0.085,0.032]	[-0.005,0.028]	[-0.042,0.057]	[-0.016,0.017]	[-0.021,0.033]
Ph.D. Student	-0.320	-1.036	0.117	-0.713	0.260	-0.267
	(0.429)	(0.089)	(0.557)	(0.139)	(0.184)	(0.457)
	[-1.115,0.474]	[-2.230,0.158]	[-0.274,0.508]	[-1.658,0.232]	[-0.123,0.644]	[-0.970,0.437]
Professor	0.136	0.197	-0.181	-0.118	-0.194	-0.198
	(0.643)	(0.693)	(0.180)	(0.775)	(0.147)	(0.355)
	[-0.440,0.712]	[-0.781,1.176]	[-0.446,0.084]	[-0.925,0.690]	[-0.456,0.068]	[-0.617,0.222]
Editor	-0.945	-0.597	0.070	-0.337	0.094	0.370
	(0.000)	(0.149)	(0.588)	(0.357)	(0.470)	(0.059)
	[-1.464,-0.427]	[-1.408,0.215]	[-0.183,0.323]	[-1.053,0.380]	[-0.160,0.347]	[-0.013,0.754]
Years in Academia	0.044	0.004	0.001	-0.012	0.002	0.002
	(0.006)	(0.891)	(0.898)	(0.649)	(0.848)	(0.899)
	[0.013,0.075]	[-0.056,0.065]	[-0.016,0.019]	[-0.063,0.039]	[-0.016,0.019]	[-0.026,0.030]
Published Papers	-0.009	-0.188	0.083	-0.140	0.136	0.255
	(0.933)	(0.149)	(0.050)	(0.239)	(0.002)	(0.000)
	[-0.207,0.190]	[-0.444,0.068]	[0.000,0.167]	[-0.373,0.093]	[0.049,0.224]	[0.129,0.380]
Written Reviews	0.319	0.036	-0.037	0.236	0.004	-0.169
	(0.003)	(0.787)	(0.335)	(0.065)	(0.920)	(0.009)
	[0.106,0.531]	[-0.224,0.296]	[-0.113,0.038]	[-0.015,0.487]	[-0.070,0.078]	[-0.296,-0.041]
Business	0.339	0.503	-0.804	-0.325	-0.058	-0.673

	(0.320)	(0.431)	(0.000)	(0.439)	(0.738)	(0.017)
	[-0.328,1.005]	[-0.748,1.753]	[-1.139,-0.470]	[-1.148,0.498]	[-0.395,0.280]	[-1.224,-0.122]
Economics	1.461	0.683	-0.808	0.599	-0.089	-0.067
and Finance	(0.012)	(0.428)	(0.000)	(0.382)	(0.660)	(0.828)
	[0.319,2.602]	[-1.005,2.372]	[-1.218,-0.397]	[-0.743,1.942]	[-0.485,0.307]	[-0.673,0.539]
Computer	0.813	-0.830	0.345	0.732	0.642	0.595
and Statistics	(0.063)	(0.141)	(0.059)	(0.236)	(0.002)	(0.025)
	[-0.043,1.668]	[-1.935,0.276]	[-0.013,0.703]	[-0.479,1.943]	[0.234,1.050]	[0.074,1.116]
Political Sciences	-0.274	-0.696	-0.514	-0.393	-0.959	-0.871
	(0.492)	(0.303)	(0.019)	(0.476)	(0.000)	(0.041)
	[-1.057,0.508]	[-2.019,0.627]	[-0.941,-0.086]	[-1.475,0.689]	[-1.376,-0.543]	[-1.704,-0.037]
Psychology	0.791	0.762	-0.637		1.044	-1.315
	(0.237)	(0.499)	(0.014)		(0.022)	(0.019)
	[-0.519,2.100]	[-1.448,2.972]	[-1.146,-0.128]		[0.421,1.667]	[-2.413,-0.218]
Sociology	0.076	0.894	-0.225	0.107	-0.506	-0.088
	(0.861)	(0.425)	(0.309)	(0.877)	(0.088)	(0.804)
	[-0.776,0.928]	[-1.301,3.088]	[-0.657,0.208]	[-1.241,1.454]	[-0.940,-0.072]	[-0.778,0.603]
Chi-Square	81.01	47.40	120.42	20.69	169.26	86.48
P > Chi-Square	0.000	0.000	0.000	0.241	0.000	0.000
Pseudo R-squared	0.117	0.142	0.051	0.048	0.069	0.076
Observations	1931	1931	1931	1935	1935	1935

Coefficients correspond to marginal effects derived from logistic regressions with p-values in parentheses and 95% confidence intervals in brackets. The number of observations is smaller than 2,222 because we only include respondents who did not select N/A in any items relevant for generating the included variables (note that the number of observations is higher than in S5 Table because we include now participants who already presented but not published papers yet). The number of observations differs between Vignette 1 and Vignette 2 because in each group two respondents in each group chose N/A options in the first vignette but not in the second vignette.
