# Supplementary material for: And the credit goes to … - Ghost and honorary authorship among social scientists
Source: PLoS One. 2022 May 5;17(5):e0267312. doi: 10.1371/journal.pone.0267312 (PMC9070929; doi:10.1371/journal.pone.0267312)
Supplement: S6 Table — (PDF) [file pone.0267312.s006.pdf]

Supporting Information for "And the Credit Goes to … - Ghost and Honorary Authorship among Social Scientists"

S7 Table. Regression results of hypothetical authorship assignments in the vignettes.

	Vignette 2			Vignette 3	
	Prof./ Postd.	Prof.	SA	Prof.	PhD
Group	15.084	-0.602	-2.632	-0.034	-0.195
	(1262.236)	(0.828)	(1.308)	(1.642)	(0.914)
Female	-0.350	-0.203	-0.306	-0.179	-0.136
	(0.463)	(0.159)	(0.293)	(0.340)	(0.181)
Anglophone	1.545	0.051	-0.046	1.196	-0.399
	(0.603)	(0.316)	(0.470)	(0.512)	(0.370)
Continental Europe	1.630	-0.792	-0.475	0.782	-0.020
	(0.597)	(0.309)	(0.469)	(0.483)	(0.369)
Developing Countries	1.290	-0.470	0.328	0.645	-0.386
	(0.755)	(0.352)	(0.493)	(0.577)	(0.413)
Age	0.008	-0.007	-0.035	-0.023	0.026
	(0.036)	(0.012)	(0.022)	(0.024)	(0.015)
PhD Student	-0.463	0.436	0.046	-0.697	0.158
	(0.703)	(0.279)	(0.465)	(0.499)	(0.307)
Professor	-0.381	-0.111	-0.151	0.150	-0.223
	(0.599)	(0.185)	(0.320)	(0.419)	(0.219)
Editor	-0.619	0.052	0.702	-0.041	-0.300
	(0.497)	(0.181)	(0.283)	(0.393)	(0.205)
Years in Academia	0.008	-0.008	0.031	0.014	-0.027
	(0.038)	(0.013)	(0.022)	(0.026)	(0.015)
Published Papers	0.013	0.143	0.176	-0.154	0.158
	(0.179)	(0.063)	(0.097)	(0.123)	(0.076)
Written Reviews	0.055	-0.015	-0.143	0.145	0.071
	(0.161)	(0.053)	(0.095)	(0.128)	(0.063)

Business	-0.119	0.009	-0.764	0.028	0.029
	(0.586)	(0.236)	(0.443)	(0.428)	(0.290)
Economics and	0.297	-0.337	0.473	0.200	-0.219
Finance	(0.867)	(0.276)	(0.433)	(0.577)	(0.333)
Computer and	0.758	0.823	0.812	0.406	0.648
Statistics	(0.867)	(0.290)	(0.398)	(0.546)	(0.362)
Political Sciences	-0.598	-1.342	-1.066	0.240	-1.265
	(0.711)	(0.303)	(0.679)	(0.622)	(0.329)
Psychology	Perfect	1.299	-0.713	Perfect	1.548
	Predictor	(0.456)	(0.692)	Predictor	(0.649)
Sociology	14.191	-0.804	0.000	0.730	-0.818
	(1301.751)	(0.314)	(0.550)	(0.803)	(0.354)
Female	0.542	0.256	0.208	0.295	0.210
X Group	(0.657)	(0.223)	(0.389)	(0.487)	(0.244)
Anglophone	-15.996	-0.473	0.020	-1.278	0.094
X Group	(1262.235)	(0.473)	(0.719)	(0.942)	(0.502)
Continental Europe	-15.408	0.023	1.050	-0.619	-0.205
X Group	(1262.235)	(0.463)	(0.701)	(0.928)	(0.498)
Developing Countries	-16.358	-0.022	0.341	-2.067	0.439
X Group	(1262.235)	(0.522)	(0.743)	(0.966)	(0.563)
Age	0.004	0.015	0.069	0.015	-0.023
X Group	(0.051)	(0.017)	(0.028)	(0.034)	(0.019)
PhD Student	-0.295	-0.394	-0.950	0.815	-0.053
X Group	(0.980)	(0.394)	(0.794)	(0.789)	(0.428)
Professor	0.574	-0.196	-0.067	-0.158	0.157
X Group	(0.839)	(0.272)	(0.440)	(0.595)	(0.299)
Editor	0.609	0.125	-0.525	0.028	0.315
X Group	(0.761)	(0.265)	(0.398)	(0.576)	(0.285)
Years in Academia	-0.036	0.018	-0.048	-0.004	0.028
X Group	(0.053)	(0.018)	(0.029)	(0.036)	(0.020)
Published Papers	-0.285	0.008	0.168	-0.065	-0.056
X Group	(0.239)	(0.091)	(0.132)	(0.180)	(0.101)
Written Revies	0.373	0.043	-0.054	0.153	0.015
X Group	(0.259)	(0.077)	(0.132)	(0.193)	(0.086)
Business	0.139	-0.157	0.117	0.357	0.481
X Group	(0.857)	(0.349)	(0.578)	(0.638)	(0.386)
Economics and	1.032	0.509	-1.196	0.855	0.299

Finance X Group	(1.439)	(0.413)	(0.645)	(0.918)	(0.444)
Computer and	0.358	-0.394	-0.402	0.434	0.294
Statistics X Group	(1.251)	(0.420)	(0.542)	(0.797)	(0.479)
Political Sciences	1.018	0.713	0.252	0.183	0.726
X Group	(1.159)	(0.435)	(0.877)	(0.908)	(0.449)
Psychology	Perfect	-0.507	-1.572	Perfect	-0.226
X Group	Predictor	(0.639)	(1.269)	Predictor	(0.810)
Sociology	-14.342	0.535	-0.197	-0.749	0.509
X Group	(1301.751)	(0.453)	(0.723)	(1.012)	(0.474)
Chi-Square	40.93	203.39	112.75	42.91	141.08
P > Chi-Square	0.162	0.000	0.000	0.116	0.000
Pseudo R-squared	0.095	0.082	0.099	0.062	0.067
Observations	1877	1935	1935	1868	1926

Coefficients correspond to marginal effects derived from logistic regressions with standard errors in parentheses. *X Group* indicates interaction terms created as the product of the respective variable and *Group*.
