# Supplementary material for: And the credit goes to … - Ghost and honorary authorship among social scientists
Source: PLoS One. 2022 May 5;17(5):e0267312. doi: 10.1371/journal.pone.0267312 (PMC9070929; doi:10.1371/journal.pone.0267312)
Supplement: S7 Table — (PDF) [file pone.0267312.s007.pdf]

Supporting Information for "And the Credit Goes to … - Ghost and Honorary Authorship among Social Scientists"

S8 Table. Comparison of Samples for Dummy Variables.

	Sample Respondents		Insensitive Respondents		Wilcoxon rank sum test	
	Number	Share	Number	Share	z	p-value
All Respondents	2222	100.00%	138	100.00%		
Female	738	33.21%	43	32.09%	0.391	0.700
Anglophone (British Isles, North America, Australia &	866	38.97%	63	45.65%	-1.661	0.097
Continental Europe	900	40.50%	45	32.61%	1.950	0.051
Developing Countries (Latin America, Africa, Southeast	300	13.50%	16	11.59%	0.677	0.500
PhD Students	204	9.18%	9	6.52%	1.117	0.264
Professors	1156	52.03%	69	50.00%	0.492	0.623
Editors	541	24.35%	33	23.91%	0.123	0.902
Business Researchers	789	35.51%	49	35.51%	0.000	1.000
Economics and Finance Researchers	291	13.10%	18	13.04%	0.019	0.985
Computer, Operations and Statistics Researcher	352	15.84%	23	16.67%	-0.274	0.784
Political Scientists	214	9.63%	10	7.25%	0.980	0.327
Psychologists	136	6.12%	12	8.70%	-1.303	0.193
Sociologists	184	8.28%	13	9.42%	-0.501	0.616
