# Supplementary material for: And the credit goes to … - Ghost and honorary authorship among social scientists
Source: PLoS One. 2022 May 5;17(5):e0267312. doi: 10.1371/journal.pone.0267312 (PMC9070929; doi:10.1371/journal.pone.0267312)
Supplement: S8 Table — (PDF) [file pone.0267312.s008.pdf]

Supporting Information for "And the Credit Goes to … - Ghost and Honorary Authorship among Social Scientists"

S9 Table. Comparison of Samples for Integer Variables.

	Sample Respondents		Insensitive Respondents		Wilcoxon rank sum test	
	Mean	SD	Mean	SD	z	p-value
Age	46.52	13.03	46.06	12.22	0.253	0.801
Academic Working Years	17.46	12.49	17.46	12.46	-0.062	0.950
Papers Published	6.40	7.74	7.44	9.87	-0.593	0.553
Reviews Written	6.84	9.37	5.89	8.41	1.712	0.087
# Authors in Last Paper	2.83	1.25	3.30	1.45	-4.187	0.000
# Contributors in Last Paper	1.16	1.64	1.55	1.92	-2.432	0.015
