# Supplementary material for: And the credit goes to … - Ghost and honorary authorship among social scientists
Source: PLoS One. 2022 May 5;17(5):e0267312. doi: 10.1371/journal.pone.0267312 (PMC9070929; doi:10.1371/journal.pone.0267312)
Supplement: S9 Table — (PDF) [file pone.0267312.s009.pdf]

Supporting Information for “And the Credit Goes to … - Ghost and Honorary Authorship among Social Scientists”

S10 Table. List of journals and societies included in the data collection.

Category	Journals / Societies	Years
Business	Academy of Management	2011-2017
	African Academy of Management	2014-2018
	American Marketing Association	2007-2018
	Asian Academy of Management	2010-2017
	Asia-Pacific Researchers in Organization Studies	2016
	Colloquium of Personnel Economics	2012-2017
	Druid	2013-2018
	European Accounting Association	2018
	European Association of Labor Economics	2016-2017
	European Group for Organizational Studies	2010-2018
	informs Society of Marketing Science	2016-2018
	International Human Resource Conference	2017
	International Journal of Industrial Organization	2010-2018
	Korean Scholars of Marketing Science	2014-2017
	Organizational Dynamics	2010-2017
	R&D Management	2017
	Research in Organizational Behavior	2009-2017
	Society of Labor Economists	2012-2017
Economics	American Economic Association	2016-2018
	African Economic Research Consortium	2015-2018
	European Economic Association	2015-2017
	Japan Economic Policy Association	2007-2016
	Latin American and Caribbean Economic Association	2013-2017
	Middle East Economic Association	2013-2017
Finance	American Finance Association	2014-2018
	European Finance Association	2018
	World Finance Conference	2013-2018
Computer Sciences	Association for Computational Linguistics	2008-2017
	Conference on Machine Learning	2008-2018
	IEEE Conference on Computer Vision and Pattern Recognition	2017

	informs International Conference	2018
Law	European Journal of Criminology	2010-2018
	European Law Journal	2016-2018
	International Journal of Cyber Criminology	2008-2017
	International Theory	2010-2017
	Justice Quarterly	2017-2018
	Texas Law Review	2016-2018
Logistics & Transport	Conference on Sustainable Urban Mobility	2015-2016
	Conference on Transport and Engineering	2016
	EURO Working Group on Transportation	2014-2017
	International Conference on City Logistics	2015
	International Symposium on Enhancing Highway Performance	2016
	Organization and Traffic Safety Management	2016
	Symposium on Transportation and Traffic Theory	2016-2017
	Transport Research Arena	2016
	World Conference on Transportation Research	2016
Operations Research	African Federation of Operations Research Societies	2018
	Asociacion Latino-Ibero-Americana de Investigacion Operativa	2014-2016
	Brazilian Operations Research Society	2010-2017
	EURO	2009-2016
	European Journal of Operational Research	2015-2018
	IFORS	2008-2014
Political Sciences	American Political Science Review	2009-2018
	Asia-Pacific Social Science Review	2014-2018
	European Political Science Association	2008-2018
	International Political Economy Society	2011-2017
	International Political Science Association	2009-2018
	Journal of Asian Public Policy	2011-2018
	Political Geography Journal	2008-2018
Psychology	American Psychological Association	2018
	Clinical Psychology Review	2011-2018
	Cognitive Psychology Journal	2007-2018
	European Society for Cognitive Psychology	2009-2011
	Psychologie Francais	2008-2018
	Psychology of Learning and Motivation Journal	2010-2017

Sociology	American Sociological Review	2009-2018
	Asian Social Sciences	2009-2018
	European Sociological Association	2013-2017
	Latin American Sociological Association	2013-2017
	South African Sociological Association	2011-2017
