# Supplementary material for: And the credit goes to … - Ghost and honorary authorship among social scientists
Source: PLoS One. 2022 May 5;17(5):e0267312. doi: 10.1371/journal.pone.0267312 (PMC9070929; doi:10.1371/journal.pone.0267312)
Supplement: S1 Text — (PDF) [file pone.0267312.s011.pdf]

Supporting Information for "And the Credit Goes to … - Ghost and Honorary Authorship among Social Scientists"

S1 Text. The text of the vignettes employed in the study.

A. *First Vignette*

A Postdoc has a research idea and proposes this to the professor. They together set up the research design. Afterwards the postdoc engages in the search for literature and summarizes the findings. Based on this the professor sets up a survey which is conducted and documented by a student assistant. The data is afterwards statistically analyzed by the professor who passes the results on to the postdoc. The postdoc then writes a journal paper which is reviewed by the professor before the submission.

B. *Second Vignette*

A Postdoc/Professor 1 has a research idea and creates the research design. This research design is then presented to the professor/Professor 2. The professor/Professor 2 revises the research design and suggests that the survey could be conducted in one of his larger classes instead of using an internet survey tool. The postdoc/Professor 1 engages in the search for literature and summarizes the findings. Based on this the postdoc/Professor 1 sets up a survey and conducts it in the professor's/Professor 2's lecture together with the help of a student assistant. The postdoc/Professor 1 then inputs the data into a statistical program and analyzes it. The results are presented to the professor/Professor 2 who gives comments about using two additional statistical tools to test for robustness and

sample bias. The Postdoc/Professor 1 includes these suggestions and writes a journal paper which is proof-read by the professor/Professor 2 before the submission.
