# Supplementary material for: And the credit goes to … - Ghost and honorary authorship among social scientists
Source: PLoS One. 2022 May 5;17(5):e0267312. doi: 10.1371/journal.pone.0267312 (PMC9070929; doi:10.1371/journal.pone.0267312)
Supplement: Attachment — Submitted filename: Response to Reviewer 3.pdf [file pone.0267312.s014.pdf]

I thank and commend the authors for the hard work of revising this manuscript. Its quality is greatly improved, and I especially appreciate how the ICMJE guidelines are re-contextualized to be less absolutist. In total, I feel that my comments and suggestions were adequately addressed, though I still have some minor issues which I believe should be addressed before submission, and which I detail below,

Dear Reviewer 3,

Thank you for your valuable efforts and comments in this and the previous review rounds. We are glad to learn that we made further progress in this round and pending some minor changes you would view the paper moving towards publication. In the due course of this revision, we paid close attention to the remaining issues that you highlight and addressed all of them in this revision. In the following, we individually respond to each of your comments:

- *The number of survey respondents is inconsistent. The abstract says 2,222, but the paper reports 2,223 in another case. Moreover, smaller numbers are used in the actual statistics analyses. I advise the authors to ensure consistency between these numbers, and make clear why numbers change throughout the manuscript. Moreover, the abstract should list the actual number of respondents used in the analysis, not the number before exclusions, so as not to overstate the size of the data being analyzed.*

Thank you. We absolutely concur with your assessment here. The sample size constitutes a crucial characteristic of every empirical study and ours is no exception. We therefore agree with your notion that it is of utmost importance that readers, and especially potential replicators, understand the composition of our sample. We explain the different reasonings behind the sample sizes in the following.

1) The confusion between 2,222 and 2,223 arose because we excluded one respondent who provided nonsensical answers to the survey (e.g. claiming to be in academia for 100 years but having only an age of 67 years).

2) In addition, you correctly point out that the sample sizes of our models differ from 2,222. This arises from the fact that only scholars having already published at least one scientific journal article could be included the first part of the paper when analyzing actual authorship assignments.

3) However, the second part of the analysis, the hypothetical assignments of authorship, could and was also be filled out by junior academics (e.g. PhD students) who have yet not published a paper. This explains why the sample size in the second part of the results exceeds the sample size of the first part.

4) Moreover, the individual models in Table 1, 2 and 3 differ from each other in terms of sample size because we excluded respondents who submitted “N/A”-answers for variables that were included in the corresponding regressions. For example, the sample size of model 1 and model 3 (ghost authorship) in Table 1 is only 1854 whereas the sample size of model 2 and model 4 (honorary authorship) in Table 1 is 1857. This derives from the fact that three respondents chose N/A options when answering ghost authorship related questions, but those three respondents did not choose N/A options when answering honorary authorship related questions. Hence, they were included in one

model but not the other. When, however, estimating the models with respondents who are eligible for all analyses (except for Model 5 of Table 1) the results remain invariant.

5) The largest sample size deviation occurs in Model 5 of Table 1. There are only 804 observations. This derives from the fact that the additionally included variable *Rate of Ghost Authors* is calculated by dividing the number of ghost authors by the number of non-author contributors. To avoid a divide by zero error, we refrained from including respondents stating that no non-author contributed to their last published paper, leading to the exclusion of 1,014 observations. As the scope of this analysis is on the extent of ghost authorship and not the occurrence, this sample size reduction is sensible.

Obviously, the responsibility to clarify these deviations rests with the authors and we realized that we have not been attentive enough in our writing to clarify these issues fully. We have rectified this in our revision and also made corresponding changes to the manuscript. Thanks to your constructive comments, we now explicitly highlight the reasons for the different sample sizes in our writing. We therefore updated multiple parts of the manuscript and the supplementary material to transparently report the number of observations used in the models. First, the manuscript now explicitly states on page 13 that the original sample size consists of 2,222 respondents. We then go on explaining why the sample size between the models differ. Hereby the updated manuscript refers explicitly to model 5 in Table 1, "Respondents stating that their last published paper did not include any non-author contributors were excluded when comparing the actual and perceived rate of ghost authors in model 5 of Table 1 because of divide by zero errors."

Moreover, Table 1 and Table 3 now state that more information on the varying number of observations can be found in the corresponding S5 Table and S6 Table. We added explanatory texts to the note of S5 Table and S6 Table outlining in detail the changes in the number of observations. We also extended the note of Table 2 explicitly stating that "The number of observations differs between Vignette 1 and Vignette 2 because in each group two respondents in each group chose N/A options in the first vignette but not in the second vignette."

- Line 50: The use of "development of technologies" here is awkward, largely because a) technology is not listed in the corresponding reference, b) it is of a different kind to the other items in the list, and c) it is unclear whether "complex nature…" refers to "development of technologies". Overall, I recommend cutting this phrase.

Thank you. We followed your suggestion and deleted this phrase.

- Line 51: When mentioning the increasing complexity of science, the authors should cite the canonical paper in this area about the Burden of Knowledge: https://academic.oup.com/restud/article-abstract/76/1/283/1577537

Thank you for pointing this out. The seminal work of Prof. Jones is highly relevant to our research. We therefore included this citation when discussing the increasing complexity of science.

- Line 70: The use of "falsify" is, while accurate, also very strong and brings to mind fraud, which I believe the authors don't want to insinuate. I suggest replacing this with a work like "distort" or

“confound” which has a similar meaning but with a less harsh connotation.

Thank you. We now write “ghost authorship and honorary authorship distort citation and publication counts.”

- Line 71: There is a double-period here, which should be fixed

Thank you. We deleted the double period.

*- Line 81: the use of the phrase “and even” in the sentence “…methodology, software, **and even** writing, reviewing, and editing* is strange and may confuse the reader—I suggest simply cutting this phrase.*

Thank you. We followed your suggestion and deleted this phrase.

- Line 113: It is unclear when reading the list of ICMJE criteria whether an author must meet one of these criteria or all of them. The ICMJE website makes it clear that the author must meet -all- criteria by appending an “AND” at the end of each list item. I suggest the authors do the same, such that the list looks like: “…acquisition of data, or analysis and interpretation of data; and 2) drafting the article…”

Thank you. This enhances the readability and the comprehensiveness of the presentation of the ICMJE authorship criteria. We originally refrained from inserting *AND* because of the direct citation. We now included an additional *AND* between the first and the second criterion.

- Line 137, in the sentence “First, pressure from co-authors, whose citation and publications counts usually benefit from a lower number of contributors can lead to researchers declining or being declined authorship;…”; while I see how credit itself (something that is abstract, social) is affected by adding co-authors, it is unclear how adding co-authors would affect the citation and publication counts of a paper, which in nearly all cases use “full-counting” to count every contribution equally. I suggest rephrasing this sentence to be more clear, or to provide a reference for how publication/citation counts would be affected.

Thank you. We agree that our statement here was misleading given the importance of full counts in the most important scientific databases like Google Scholar or SCOPUS. We therefore rephrased this part and included a separate sentence elaborating on the increase in usage of fractional counts and its implications on scholarly productivity. We hereby refer to the literature review of Waltman (2016).

- Line 165: I appreciate the authors adding in the chaperone effect, but I think the current description is a little misleading. The Chaperone Effect is not really about repetitional bias in peer review at elite journals, as the authors claim here. Rather, it refers to a kind of mentorship, where a senior author guides their student in how to write for a specific, elite, journal. If the authors would like a reference about the bias towards famous authors, I recommend this by Tomkins, Zhang, & Heaviln (2017):

https://www.pnas.org/content/114/48/12708

Thank you. We deleted our reference to the Chaperone Effect. Instead, we include now Tomkins et al. (2017) to support the argument.

- Line 203: I appreciate that the authors added a mention of the Matilda Effect which I mentioned, but I think that the description provided misses a key nuance—it is not only that women receive less credit for their work, but that the credit tends to go to male colleagues involved in the study. This can be potentially damaging in the case of honorary authorship, in which adding honorary senior (often male) colleagues can "steal credit" away form women in the team.

Thank you. We clarified this sentence. We now write: "Female researchers are arguably among the most vulnerable, as credit often tends to go primarily to their male colleagues."

- Line 581: The authors should be more specific with "Anglophone", "Central European", and "Asian" scholars. Specifically, there are likely many Anglophone scholars in Central Europe, and Asia scholars in North America and Europe. As such, referring to these groups by their ethnicity can be misleading, as an Korean scholar residing in a Canadian university is likely to face very different authorship pressures than if they were in their home country. My suggestion is to rephrase to say something like "scholars residing in Anglophone, Central European, and Asian countries…".

Thank you. We completely agree that it is important that we refer to scholars living (and working) in those areas. We therefore went through the whole manuscript and changed all corresponding phrases to scholars residing in Anglophone, Central European, Asian, … countries.

- Regression tables: The authors should mention the reference level for each categorical variable, either included directly in the table (as a named row with no coefficients) or in the table caption. For example, it is clear that "Male" is the reference level for the gender variable, but it is not clear what the reference level for geography is, or job title.

Thank you. Reporting our results as transparent as possible is very important to us. We therefore added the following sentence to the regression tables: "The (omitted) reference categories for the categorical variables are *Asia* for geographical region, *Postdoc* for job position and *General Social Sciences* for research field."

- Fig 1: The decimal points for percentages on the Y-axis can be removed to make the graph more clean

Thank you. We deleted the decimal points.

- Fig 1: An optional suggestion—making the styles consistent between figures 1 and 2 could really make them more aesthetically pleasing and make the final manuscript look more professional.

Thank you. We changed Figure 1 and Figure 2 based on your suggestions and now use the same format and colors in both figures.

- *Fig 2: text is too small and difficult to read, the font-size should be increased as much as possible*

Thank you. We increased the font size to make the legend easier to read.

- *Fig 2: the graph would be easier to read if there were borders around the bars that clearly delineated them from each other.*

Thank you. We now include borders around all bars in all figures.

- *Fig 2: This is only a suggestion, but this plot could be more readable and impactful if the authors find a way to combine A & B into a single panel, along with C & D into a second panel. For example, the bars for identified share of ghost authors could be shown, and a single point, or a line, to mark the perceived share. There are several ways this could be done, it is simply that comparing histograms side-by-side is more difficult than comparing within a single panel.*

Thank you. We changed the reporting style from a histogram to a barplot for each 10% of the share of ghost/honorary authors. This enables us to report bars for actual and perceived shares right next to each other and eases the comparison for the readers.

- *Supplemental texts: It wold be preferred to upload the supporting information as a .pdf rather than a .docx. .pdfs are more universal, so by using it you are ensuring that the files are more accessible to whomever need them.*

Thank you. We changed the format of the supporting information to PDF.
