# Supplementary material for: And the credit goes to … - Ghost and honorary authorship among social scientists
Source: PLoS One. 2022 May 5;17(5):e0267312. doi: 10.1371/journal.pone.0267312 (PMC9070929; doi:10.1371/journal.pone.0267312)
Supplement: Attachment — Submitted filename: PONE- PR OCT 2020.docx [file pone.0267312.s012.docx]

PONE-D-20-30365

This paper presents a survey study on gift and ghost authorship in the social sciences. Before speaking to the content of the paper there is the research ethics review situation. According to the authors, this study did not obtain approval for the survey study. The authors explain that social science studies do not requires IRB approval in Germany. Does PLOS allow this justification? If so, I suggest that authors could make explicit the policies (regulations) or institutional norms that protect human subjects (e.g. federal data protection, confidentiality mechanisms, informed consent).

Given the limited literature regarding authorship in this field, this paper does present some interesting empirical findings regarding rates of honorary and ghost authorship a as well as power and gender. Although I do believe that such results should eventually be published the paper still has important shortcomings.

**Major issues:**

1. There is a generalized vagueness throughout the paper which makes it difficult for the reader to follow. For example, it is unclear how multiple authors create better quality and higher productivity papers. It is also unclear why the simple presence of multiple authors gives way to malpractice. There was questionable practices before multiple authorship. This is further developed in the specific issues section below. A thorough review of the English may also help.
2. The authors often state that there is no applicable authorship criteria in social sciencs (e.g.p.30) However, there is authorship guidance in various professional societies (e.g. American Sociological Association Code of Ethics and the American Psychological Association Code of Conduct). Moreover, the Committee of publication ethics does have some guidance on authorship; <https://publicationethics.org/authorship>. Lastly, certain journals, research centers as well as funding agencies have developed some guidance that apply to social scientists
3. Surprisingly, even though the author says there is no criteria, the authors seem to explain that there is a correct and incorrect way to distribute authorship. Even in the abstract, the authors seem to want to evaluate if scholars assign authorship “correctly” in three hypothetical situations. However, if there is no criteria how could there be a correct and incorrect way to distribute authorship.
4. The recommendations at the end of the paper (p.27) do not logically follow from the actual results of the survey. For example, the author mention the importance of contributor declarations but never explains why this would be important. If a person lies about authorship they could also simply lie about contributorship. Also, ghost authors will not be listed as contributors.
   a. The authors also mention that there are power dynamics and thus whistleblowing should be used to go outside the system and change past submissions. Why is whistleblowing used to counter power-dynamics as opposed to other methods? Moreover, changing the past submission would have a huge impact on science. Not only would authors change on past papers, so would the citations and refences. Do we also take tenure away from people after the fact? Feasibility and cost would be a serious consideration. This is almost a completely different paper and argument.

Minor comments:

In many countries, the notion of malpractice is often a legal term. Is this term found in Germany in policy? To my knowledge authorship regulations are not found directly in legal documents. It may be lack of compliance, misbehavior or questionable research conduct.

p.3 l. 42 There are statements that are often vague and not explained. For example, the first sentence “Publish or Perish” epitomizes the academic reward system across scientific disciplines nowadays better than ever before”. Why is this notion more important now as compared to before? When did this shift happen?

p.54-56; sentence non-sensical. I am not sure what is the gold standard in this sentence. Often we say peer-review is the gold standard. I have not heard this about citation and publication counts being the gold standard.

p.4 l.61 As the authors mention, there is a lot more literature in life sciences and medical science when compared to social sciences. However, the author should also cite the literature that does exist regarding the rise of co-authorship in social science (1). The author can also look at studies on interdisciplinarity that include a cross-disciplinary lenses(2–8).

p. 4 l. 63 author suggest that contributor declarations have not been included in social science journals. There are no citations and it is not a commonly accepted fact. There are journals of an interdisciplinary nature that include social science work (e.g. PLOS). Some social science journals may be gathering the information about contributions during paper submissions without actually publishing the content. Also, if there are no citations to illustrate this fact. It would be relevant to exemplify this point by citing journal policies.

p.6 l. 103 ; please explain Harbring and Irlenbush study. This is not clear.

p.10 l. 183; I am unsure what the antepenultimate and penultimate task would be. What is the “last task”. Is this the one that takes the least amount of time? The most value? The idea? The methods? The manuscript writing?

p.11 203-205 ; unclear

l. 206=209 ; what is collaboration on eye level?

l.213 – workload shift should be division of labor.

p.25 l.444-447. I am unsure what result leads to the notion that not everyone knows what constitutes authorship. There may simply be differences in interpretation without the individuals not understanding the concept.

p.25 l.448-452. The author mentions objective authorship criteria. Although there are regulations in which individuals must have substantially contributed to the research to be included as author. The notion of “substantial contribution” is an ongoing debate. Thus it remains a subjective notion. If the author believes there is an objective notion at play, please explain.

p.26. Although Weinstein may have had an impact on this context, there is also increased awareness regarding gender inequities in science. The author should explain the negative impact of this inequities by referencing the literature on the subject.

p.27 l.475; the authors state that researchers from Asian countries have stronger likelihood of obedience. As written it is construed as stereotyping. Can you refer to the literature to explain the hierarchy in certain groups?

p.26. l. 444 “This accords to the authorship guidelines of their academic societies: Articles based on dissertations should always include the PhD students as authors” Can you cite these academic societies. Is this also the case in university regulations?

p. 26 I am not sure what the author considers as elderly authors. Is this actually based on age? Is is based on academic seniority? The notion “elderly” has a very negative connotation and should be avoided.

p. 26. When talking about gender please use the notion women/men. Gender is a social construct. Avoid the notion of male/female that refers to sex as a biological variable.

p. 29 the authors mention a difference between authorship and effort which is certainly true. They conclude that because there is a difference, research institutions should rely on other estimates. What are other estimates and why and how would one include the notion of effort in evaluation?

p.31l. 554 I am unsure what expressiveness implies in this context.

Methods and Results: please ensure that a second peer-review looks over statistics. I am not a quantitative researcher.
