# Supplementary material for: And the credit goes to … - Ghost and honorary authorship among social scientists
Source: PLoS One. 2022 May 5;17(5):e0267312. doi: 10.1371/journal.pone.0267312 (PMC9070929; doi:10.1371/journal.pone.0267312)
Supplement: Attachment — Submitted filename: ResponseToReviewer2.docx [file pone.0267312.s013.docx]

*Reviewer #2: This paper reports results of a survey of authors of social sciences articles showing that ghost and honorary authorships occur in the social sciences, and correspondingly suggests that authorship standards should be implemented in the social sciences, much as they already are in certain journals in the hard sciences.

Overall, the paper is well done.*

Dear Reviewer 2,

Thank you for your time and effort reviewing our manuscript. We were glad to learn that you found the topic to be timely and interesting. We were enthused to hear you found that “overall, the paper is well done.” We highly appreciate the encouraging and motivating statement.

Moreover, we are grateful for your constructive criticism and the options you laid out for us, especially regarding vignette 3 and the data availability. Accordingly, we reworked our submission and hope that the revised submission comes up to your expectations.

Below you find our responses to your comments and explanations as to how we have included them in the revised version of the manuscript.

*The one issue I see is with vignette #3. As written, this vignette mingles the authorship issues with a severe ethical issue. In this vignette “After the successful graduation of the PhD student the professor creates a scientific paper based on the empirical results of the thesis and submits it to a journal.” The vignette does not state if the masters student was listed as an author. It does not state if the masters student gave permission for the professor to turn the thesis into a paper. While the professor provided input to the study and thesis, the vignette makes it look like the professor is appropriating the work of the student. If this is indeed the case, then I have a hard time trusting the results of the survey for this vignette because it mingles the ethical issue (appropriation of work) with the authorship issues. I would imagine that many of those completing the survey would have had the same reaction, and that their answers regarding authorship would have been clouded by the ethical issue.

Given the potential confounding of vignette #3, I would suggest it be removed from the paper unless the authors can make a convincing case that no confounding occurred. In my view, removal of this vignette would not really weaken the conclusions. If the results of this vignette are removed, it would still be worth keeping something about it in the paper to highlight the ethical issue.*

Thank you very much for this comment. We highly appreciate your sentiments. We have wrestled among the authors how to best proceed. In our view, the comment relates to two underlying factors. First, you rightly point out that the wrongful appropriation of the Professor mentioned in the vignette clearly presents both an ethical violation and an authorship issue. Not mentioning someone who has contributed to a paper, is essentially both an authorship issue and the misappropriation of someone else´s work (plagiarism). Second, the third vignette does also not perfectly relate to the first and second vignette as it poses a very different scenario (PhD thesis) compared to the more “traditional” research projects described in the first two vignettes.

Considering that due to the rework we also had to extend the manuscript, we followed your notion and deleted the third vignette as well as its results from the manuscript. Nevertheless, the discussion still includes elaborations on gender effects based on our findings from the empirical study.

*Regarding data availability, I would suggest that the authors add a table (or supplement) with the list of journals/conferences associated with the papers/authors who answered the survey. The list of sources is important and cannot be considered confidential. Also, I would suggest the authors do what is necessary to fully anonymize the survey data and to make it available. Although they say “available upon request (with caveats)”, in practice this tends to simply delay the availability question and in most cases authors then decline to share data. I think data availability must be addressed before the paper is published.*

Thank you for this important notion. Having conducted research on open science and open data ourselves, we agree that it is of utmost importance to be as transparent as possible when it comes to sharing scientific knowledge.

Nevertheless, we face the issue that our dataset contains sensitive information because it allows to identify papers containing ghost and/or honorary authors. Quite frankly, we would like to avoid a situation where others might be able to potentially identify those who engaged in misconduct. Similarly, we would like to preserve anonymity of our respondents on this very sensitive topic.

In our case, certain sets of combinations of demographic, job position and productivity variables employed in the analysis might allow the indirect identification of individuals (e.g. a high prolific scholar in a specific field living in a specific region and being so and so many years old might point towards a specific researcher). Of course, we refrained from collecting any direct personal data to ensure respondents’ anonymity. However, Art. 4 of the General Data Protection Regulation (EU) 2016/679 (GDPR) states that also data that might lead to indirect identifications of persons is considered as personal data (and thus must not be made publicly accessible). Therefore, we unfortunately cannot make the data publicly available according to GDPR and the anonymity we promised respondents who filled out the questionnaire.

Nevertheless, we managed to find a solution that accommodates your request and assuages the privacy concerns. In case you (or others) are interested, the Data Protection Officer at Bern University of Applied Sciences will provide access after the signing of a non-disclosure agreement. Contact should be direct to: datenschutz@bfh.ch.

We hope this information better explain the sensitive nature of our data and how we would be able to make the data available via the institutional Data Protection Officer.

.
